# Pathological Observations on the Brain

**Published:** 1790

**Authors:** Thomas Anderson

**Affiliations:** Surgeon at Leith, and Fellow of the Royal College of Surgeons


IX. *Pathological Observations on the Brain. By Mr.* Thomas Anderson, *F. R. S. Edin. Surgeon at Leith, and Fellow of the Royal College of Surgeons.— From The Transactions of the Royal Society of Edinburgh,* Vol. II.

THE following observations may serve to illustrate and confirm the opinion now very generally adopted by anatomists and physicians, that an affection of one hemisphere of the brain, whether from internal disease or external accident, produces its morbid symptoms on the opposite side of the body.

CASE I.

A lady, about forty years of age, whom I attended along with Dr. Monro, was for many years affected with violent head-achs; she complained of the pain being most violent in the crown of her head, which at last brought on convulsive tremors of the left arm and leg: these often continued half an hour, and would return three or four times a day; the fits grew more severe and frequent, and the right side became affected, and frequently she was comatose tofe for twenty-four hours, till, quite worn out, ſhe died in November, 1770.

On opening her head, when the dura mater was taken off, on the right hemiſphere of the brain, there was a loſs of ſubſtance for about two inches and a half in length, one and a half in breadth, and about the middle near an inch deep, the length of which was in the direction of the falx: in the middle of this, immediately under the coronal ſuture, and on the ſide neareſt to the falx, within an inch of it, there was ſome ſoft browniſh matter in the bottom, on touching of which with the knife I diſcovered ſtony concretions, which were taken out and waſhed. Several of them broke into ſand on the ſlighteſt touch; but four or five of them, each about the ſixth of an inch in length and breadth, and a little thicker than the ſhell of an egg, I ſaved, and afterwards gave to Dr. Monro, who was not then preſent.

### CASE II.

William C., aged about forty-five years, of a corpulent habit, was, for ſeveral years, ſubject to epileptic fits, which commonly returned every three or four weeks, and any irregularity in eating or drinking would immediately bring them them on; but when cautious, living ſparingly, and taking ſome laxative, he was often free from them for four or five months.

The fits always came on with convulſive motions in the right arm and leg, which, in a few minutes, were ſucceeded by ſtupor, in which he continued above half an hour. In November, 1775, he received a ſtroke on his head, which brought him to the ground; was inſtantly ſeized with one of the fits; and in twenty-four hours had ten or twelve of them, in all of which the only parts convulſed were the right arm and leg; the fits became more frequent, a total ſtupor came on, and he died fourteen days after.

On opening his head, on the left hemiſphere, immediately under the coronal ſuture, and an inch from the falx, the dura mater adhered to the brain for about the ſize of a ſhilling, and was ſo much thickened and hardened as to be in a cartilaginous ſtate; the brain, for the ſize of a large walnut, was much hardened, and the under part of it adhered ſlightly to the falx; on the outer ſide of this hardneſs, on that ſide fartheſt from the falx, and in the middle of the ſubſtance of the cerebrum, there was about an ounce ounce and a half of extravaſated blood, which was ſoft, and of a black colour.

### CASE III.

Robert H., a ſailor, aged about forty years, when on board of ſhip, ſtooping down, received a violent ſtroke on the back part of the parietal bones by the falling of a boom; there was no wound, but the parts were much bruiſed. Some months after he complained of a pain immediately under the part on which he received the ſtroke, which gradually grew worſe, and in a year and a half the pain was moſt excruciating, and brought on violent convulſions in both upper and lower extremities of both ſides, the violence of which, in ſome months, put an end to his life.

On opening the head, the poſterior part of both hemiſpheres of the brain was found greatly inflamed and much hardened, and adhered firmly to the dura mater and the falx; the left ſide was more diſeaſed than the right, and the dura mater, in ſome places where it adhered firmly, was much thickened, and almoſt cartilaginous.

### CASE IV.

Mr. L., by a fall down a ſtair, fractured the left parietal bone. I ſaw him in half an hour, when he was in a ſtupor. He was immediately bled very plentifully, and then carried home. The fracture extended from the middle of the bone downwards and backwards, and was traced near to the maſtoid proceſs; but I could not carry the inciſion any farther. A piece of the bone was taken out by the trepan; a conſiderable quantity of extravaſated ſerum and blood was found preſſing on the dura mater, which was got out; the wound was dreſſed, and he was bled very plentifully a ſecond time; after which he became ſenſible, and anſwered diſtinctly when ſpoken to, and, after ſleeping ſome hours, was greatly relieved, but at times the right leg and arm were attacked with convulſive tremors, which continued for three days, and on the fourth day every ſymptom appeared very favourable, and he had the appearance of doing well; but he frequently complained of a pain in his head. On the twentieth day he was ſeized with rigor, which was ſucceeded by a feveriſh paroxyſm, that frequently returned for three days: his pulſe became conſtantly quick, and he died on the twenty-eighth day. His friends would not confent to his head being opened.

### CASE V.

Alexander H., a lad of eighteen years of age, fell into the hold of a fhip about fifteen feet down, and was carried home in a ftupor. A tumefaction was obferved on the top of the right parietal bone. After a plentiful bleeding he recovered of the ftupor; a laxative was given, and he was bled again in the evening. On the third day the ftupor returned; and on the evening of that day I was called in, when there was every fymptom of compreffion of the brain; and next morning Dr. Monro and Dr. Auftin were fent for. It was then judged proper to examine the ftate of the right parietal bone, where the tumefaction was at firft obferved. No fracture could be found; but a piece of the bone was taken out by the trepan. Nothing was feen that could occafion any preffure. The ftupor, &c. continued, and he died the thirteenth day. Eighteen hours after his death I went to open his head; but fuch a degree of putrefaction was come on, that a great part of the brain had come out of the hole in the bone, quite diffolved and putrid. The teguments were taken off, but no fracture was found in any part of the head.

### CASE VI.

A failor boy, of fourteen years of age, fell into the hold of a fhip. He was carried afhore in a ftupor. There was a fwelling on the middle of the right parietal bone, without any wound. He was bled, and put to bed, and in half an hour was fo much recovered, that it was thought unneceffary to infpect the ftate of the bone. He was ordered a laxative to take in the night, but next morning it had not operated. It was then repeated, and in the evening he appeared very well; but there feemed to be a degree of torpor in the inteftinal canal, from the laxatives not operating. A clyfter was given, and the laxative again repeated. Next morning his left arm and leg were quite paralytic, the pupil of the left eye was dilated, and did not contract when a lighted candle was brought near it, nor was he fenfible of its being there; but he could read diftinctly with the other eye, and the right leg and arm were very well. In the afternoon, juft forty-eight hours from the time that he met with the accident, the bone was was laid bare, and in the middle of the right parietal bone a piece was found to be broken off more than an inch ſquare. The upper ſide had pierced the dura mater, and gone into the ſubſtance of the cerebrum. The broken piece was eaſily taken out, and the wound dreſſed. Immediately after the pupil of the left eye contracted, and he could diſtinguiſh large objects with that eye, and the leg and arm were leſs affected. He had a good night, and next morning could read when the right eye was ſhut. On the third day after the operation, when the wound in the dura mater inflamed, and a conſiderable tumefaction came on, his left eye, leg, and arm, became again paralytic, with frequent convulſions in the left leg and arm, but without the ſmalleſt complaint in the other ſide. In this ſtate he continued for ſeveral days; a ſuppuration came on, the ſwelling went off; after which he continued well, and the wound healed up in eight weeks.

From theſe caſes I ſhould infer,

1. That when one hemiſphere of the brain is affected, it generally produces its morbid ſymptoms on the oppoſite ſide of the body.

2. That

2. That when both hemifpheres are affected, the whole body fuffers.

3. That though one hemifphere only is affected, when the injury is great, the whole body will fuffer.

4. That though the cerebrum alone is hurt, it produces morbid fymptoms in all mufcles of voluntary motion, whether their nerves take their rife immediately from the cerebrum, from the cerebellum, or from the medulla oblongata.

5. That, in cafes of external accident, where one fide is affected, it is more favourable than when both fides fuffer.

